# Primary Mucosa-Associated Lymphoid Tissue Lymphoma of Thyroid with the Serial Ultrasound Findings

**DOI:** 10.1155/2016/5608518

**Published:** 2016-03-23

**Authors:** Eon Ju Jeon, Ho Sang Shon, Eui Dal Jung

**Affiliations:** Department of Internal Medicine, Catholic University of Daegu, School of Medicine, Daegu 42472, Republic of Korea

## Abstract

Extranodal marginal zone lymphoma of mucosa-associated lymphoid tissue (MALT) of the thyroid gland is uncommon. Even though its natural history is not well defined, it is known to be indolent course. We present a case of primary MALT thyroid lymphoma with the serial sonographic findings in the patient presenting as the focal nodule. A 45-year-old woman visited our hospital for neck examination. Initially, fine-needle aspiration cytology in the focal hypoechoic lesion in the left thyroid lobe on ultrasound sonography was performed and consistent with Hashimoto's thyroiditis. However, the results of serial ultrasounds and core-needle biopsy revealed an extranodal marginal zone lymphoma of MALT on 4-year follow-up. Patients with a focal hypoechoic nodule with linear echogenic strands and segmental pattern in the background of Hashimoto's thyroiditis on ultrasonography should undergo careful surveillance for malignancy. Serial sonographic features in this case are meaningful in the understanding of the natural history of the extranodal marginal zone lymphoma of MALT of the thyroid.

## 1. Introduction

Primary thyroid lymphoma is a rare thyroid tumor, accounting for approximately 2–8% of thyroid malignancies and for 1-2% of extrathyroid lymphomas [1–3]. The most of thyroid lymphomas are non-Hodgkin's lymphomas of B cell origin. The most common subtype is diffuse large B cell lymphoma (DLBL) and the other subtype is an extranodal marginal zone lymphoma of MALT comprising 6% to 27% of thyroid lymphomas [1]. Extranodal marginal zone lymphoma of MALT is rare and it can occur in a variety of organs, including the orbit, conjunctiva, salivary glands, skin, thyroid glands, and lung even though the most common sites are stomach and intestine [2]. These tumors are often localized and of indolent clinical behavior.

Because thyroid gland has no native lymphoid tissue, thyroid lymphoma is thought to develop from chronic autoimmune thyroiditis such as Hashimoto's thyroiditis [1, 4, 5]. In addition, because thyroid gland is not a mucosal organ, extranodal marginal zone lymphoma of MALT of the thyroid is thought to be common occurring in chronic autoimmune thyroiditis. However, the mechanism of underlying malignant transformation has yet to be clearly elucidated.

For the evaluation of the thyroid nodule, fine-needle aspiration cytology (FNAC) is relatively accurate test and can be easily performed. Nevertheless, the diagnosis of thyroid lymphoma using FNAC or core-needle biopsy (CNB) is difficult. Therefore, there are often cases that are diagnosed with immunohistochemical stating after surgery.

Although there are several studies about extranodal marginal zone lymphoma of MALT accompanied with Hashimoto's thyroiditis, we herein report the experience with the serial ultrasound findings and FNAC results of the course of primary MALT thyroid lymphoma in the patient with focal nodule and Hashimoto's thyroiditis.

## 2. Case Report

A 45-year-old woman visited our hospital for neck examination in March 2010. The mild goiter had been present over several years. However, she does not have further evaluation about it. In the meantime, there is no significant change of the goiter. There were no symptoms such as dyspnea, hoarseness, pain, and the difficulty of swallowing. There was no change of weight, fever, or night sweats. She had no history of thyroid disease or radiation exposure. She did not smoke and had no familial history of endocrine disorders. On physical examination, the thyroid was diffusely enlarged and there is no palpable nodule and focal tenderness on neck. There is no hepatomegaly and splenomegaly on abdomen. Her physical examination was otherwise unremarkable. Her blood pressure was 120/70 mmHg and body temperature was 36.8°C.

The laboratory findings were as follows: thyroid function tests: free T4, 0.864 ng/dL (normal range, 0.8 to 1.9 ng/dL); T3, 1.08 ng/dL (normal range, 0.6 to 1.7 ng/mL); thyroid stimulating hormone (TSH), 7.89 *μ*IU/mL (normal range, 0.4 to 4.7 *μ*IU/mL); antithyroglobulin antibody level increase, 1 : 25; antimicrosomal antibodies, negative. Subclinical hypothyroidism was suspected.

**Figure 1:**
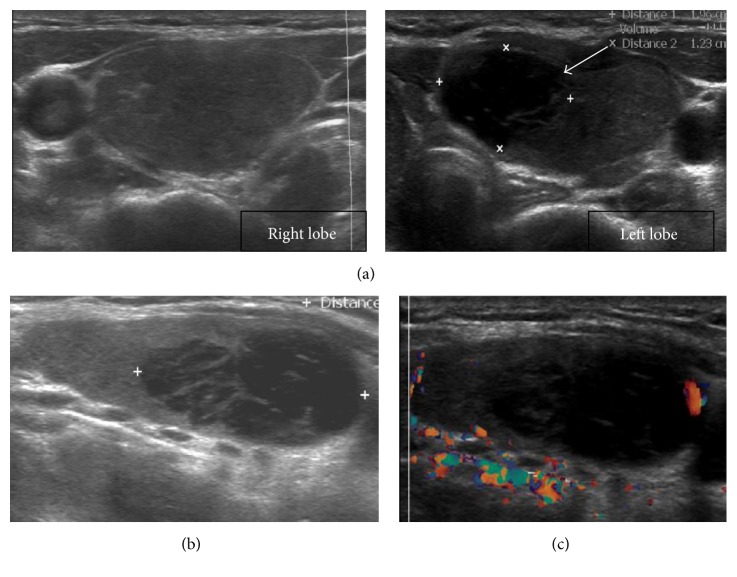
(a) Thyroid ultrasound shows a markedly hypoechoic area (arrow), with interspersed linear echogenic strands pattern in transverse view and (b) longitudinal view. (c) Color Doppler image shows a left thyroid nodule with peripheral increased blood flows in longitudinal view during a first visit.

**Figure 2:**
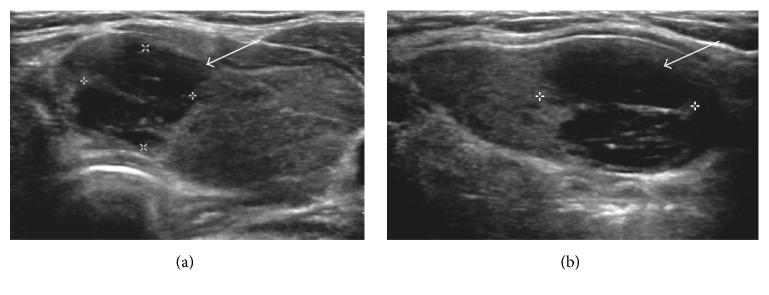
Four years later, image of the left thyroid nodule (a) in transverse view and (b) longitudinal view.

**Figure 3:**
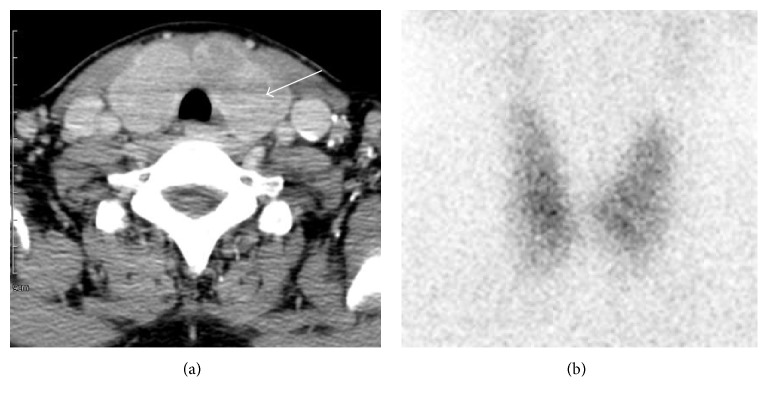
(a) Neck computed tomography shows 1.9 × 1.2 cm sized hypodense mass involving thyroid isthmus in the left thyroid. (b) Thyroid scan with Tc-^99^m pertechnetate shows diffuse and even uptake in both thyroid glands.

Initial ultrasonography (US) revealed diffuse enlargement of both thyroid glands, with heterogeneous background parenchyma and a focal hypoechoic nodule measuring 1.9 × 1.2 × 2.4 cm in upper portion of the left thyroid gland (Figure 1). US-guided fine-needle aspiration cytology (FNAC) showed the presence of lymphocyte. There were no malignant findings. Therefore, the nodule was suspicious of the lymphocytic thyroiditis lesion. Six months and one year later, US-guided FNAC was followed. There were no significant changes of the size and finding on US. The results of FNAC showed lymphocytes repeatedly. However, on follow-up four years later, the results of FNAC showed atypical lymphoid proliferation in February 2014 (Figure 2). US-guided CNB was performed, which showed thyroid follicular atrophy and lymphocyte infiltration in the interstitial tissue. In the immunohistochemistry stain, CD20 was positive and CD5 was negative. The histological findings were suggestive of B cell lineage. Cervical contrast-enhanced computer tomography (CT) showed a low-density nodule in the same area and benign or intermediate nature lymph nodes in levels of lb and lla both of the lateral neck (Figure 3(a)). Thyroid scan showed diffuse and even uptake (Figure 3(b)). All the hematological and biochemical investigations were within the normal range. She underwent total thyroidectomy and lateral neck node dissection. The specimen included the 6.5 × 4.5 × 3.5 cm left thyroid lobe and 7.2 × 3.3 × 3.0 cm right thyroid lobe. The tumor size was 1.5 × 1.0 cm.

**Figure 4:**
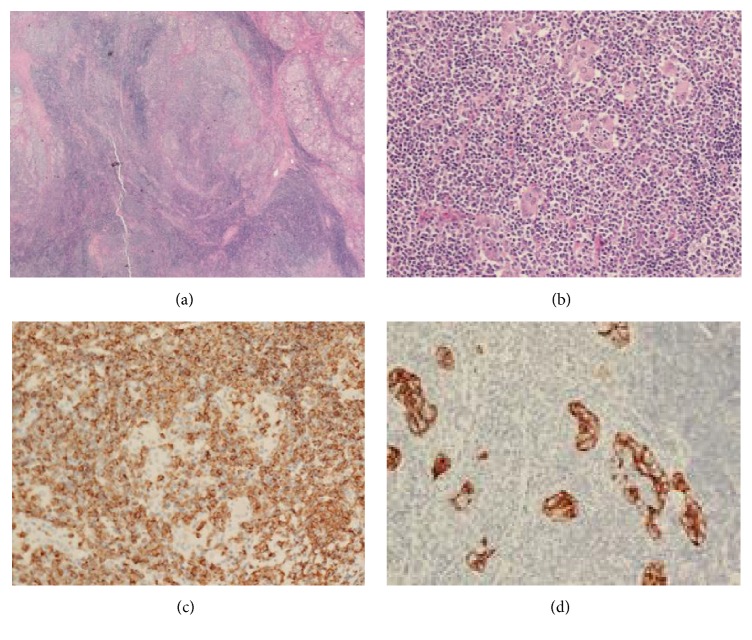
(a) Microscopic finding shows the extranodal marginal zone lymphoma of MALT in a background of Hashimoto's thyroiditis (H&E stain, ×20). (b) Microscopic finding of the thyroid mass shows marked lymphocytic infiltration and a few degenerated follicles (H&E stain, ×200). (c) Immunohistochemical findings disclose diffuse positive staining for CD20 (immunohistochemistry, ×200). (d) Immunostaining for cytokeratin highlights lymphoepithelial lesions (immunohistochemistry, ×200).

On microscopic examination of the tumor margin, the findings showed diffuse lymphocytic infiltration in the normal thyroid parenchyma, thyroid follicular atrophy, and the formation of the lymphoid follicles (Figure 4(a)). This was compatible with Hashimoto's thyroiditis. The tumor was diffusely replaced by an atypical lymphocyte with small and centrocyte-like cell. These cells gave rise to numerous lymphoepithelial lesions (Figure 4(b)). The metastasis of central neck node was not evident. Immunohistochemistry showed CD20 positivity, CD5 negativity, and cytokeratin positivity. She was diagnosed with the MALT lymphoma (Figures 4(c) and 4(d)). Postoperative ^18^F-fluorodeoxyglucose positron emission tomography of the neck performed and revealed the uptake in the cervical, axillary, and iliac lymph nodes. Using the Ann Arbor staging system, she was at stage IIIE, and R-CHOP chemotherapy was considered.

## 3. Discussion

Primary thyroid lymphoma is a rare thyroid tumor, and most of them are DLBL of B cell origin. Extranodal marginal zone lymphoma of MALT of the thyroid has been reported to vary between 6 and 27% according to the literature [1]. Primary thyroid lymphoma is usually occurring in patients with Hashimoto's thyroiditis. Extranodal marginal zone lymphoma of MALT has an indolent course, while DLBL has the aggressive progress. The diagnosis of the extranodal marginal zone lymphoma of MALT is often delayed because systemic symptoms do not appear well.

Thus, although lymphoma of the thyroid gland is relatively rare, there are a few studies. However, the development of an extranodal marginal zone lymphoma of MALT has been linked to chronic autoimmune disease such as Hashimoto's thyroiditis and it is interesting. In the normal condition, thyroid gland is an organ that lacks the lymph nodes; however, if the autoimmune disease is accompanied, it acquired the infiltration of lymphocytes into lymphoid tissue [5–7]. B cells are present and the differentiation of plasma cells appeared in this tissue. That is very similar to MALT. DLBP is present in the transition between the DLBL and the extranodal marginal zone lymphoma of MALT, and DLBL is thought to be rapidly converted from an extranodal marginal zone lymphoma of MALT. In Korea, since Lee et al. reported an extranodal marginal zone lymphoma of MALT at first [8], it has gradually been known about the clinicopathologic characteristics [9, 10]. Most had a history of long-term Hashimoto's thyroiditis. Some did not accompany Hashimoto's thyroiditis. It was reported that de novo extranodal marginal zone lymphoma of MALT may occur without the preceding thyroiditis. Most of cases have an abrupt change in size and compressive symptoms were developed by mass effects.

In the diagnosis of the thyroid lymphoma, it is important that clinicians suspect it. Most patients with thyroid lymphoma showed rapidly increased goiter between 1 and 3 months at about 70%, and in approximately 30% the patients complained of obstructive symptoms such as hoarseness [1, 3, 11, 12]. It was involved with unilateral lobe or both lobes of thyroid. It was hard and there was no pain. Therefore, in patients with Hashimoto's thyroiditis, extranodal marginal zone lymphoma of MALT should be suspected if thyroid grows rapidly and they had compressive symptoms between the weeks or months. The most common cause of sudden increases for differential diagnosis is bleeding of benign tumor of the thyroid gland. However, in this case, the patient mainly complained of pain and it usually disappears in a few days. If the goiter becomes large rapidly causing pressure symptoms, it should consider the possibility of anaplastic cancer or thyroid lymphoma. Typical symptoms of lymphoma such as fever, night sweat, and weight loss are rare [3]. Extranodal marginal zone lymphoma of MALT is slowly proceeding and tends to remain localized for long time.

Even though this patient had no symptoms except stable bulging neck, she had a medical checkup by chance and was diagnosed with subclinical hypothyroidism due to Hashimoto's thyroiditis and focal nodule. Initially, FNAC in the focal hypoechoic lesion in the left thyroid lobe on US was performed and consistent with Hashimoto's thyroiditis. However, the results of serial USs and CNB revealed an extranodal marginal zone lymphoma of MALT on 4-year follow-up. Recently, thyroid nodules even if they are not palpable are detected easily with the spreads of US and US-guided FNAC is available. Therefore, the diagnostic accuracy of the first-line US and FNAC of an extranodal marginal zone lymphoma of MALT of the thyroid compared with these of differentiated thyroid cancer is low and varies widely. Considering that there was no change in thyroid lesions on sequential US follow-up, this case is a possibility that the diagnosis had been delayed due to the limits of FNAC compared to the insidious onset of it. In general, the US findings that suggest thyroid malignancy are well known as follows: marked hypoechogenicity, irregular margins, and a taller-than-wide shape. However, the characteristic US features of the extranodal marginal zone lymphoma of MALT are not known well. At present, Orita et al. reported characteristic US features of MALT lymphoma of the salivary and thyroid gland [13]. Mainly, two US patterns were observed for the extranodal marginal zone lymphoma of MALT: the interspersed linear echogenic strands pattern and the segmental pattern. In this patient, US feature was similar to linear echogenic strands pattern that are considered to be fibrous bands (Figures 1 and 2).

In the case of an extranodal marginal zone lymphoma of MALT accompanied with Hashimoto's thyroiditis, the pathological and immunohistochemical examination of the tissue is required to confirm it because of coexisting neoplastic lesions and reactive lesions [6]. Even though there are no specific markers, extranodal marginal zone lymphoma of MALT can be diagnosed immunohistologically, by the presence of immunoglobulin light chain, CD20, and Bcl-2 and the absence of CD5, CD10, and CD23.

The prognosis of the extranodal marginal zone lymphoma of MALT localized to the thyroid is excellent and the 5-year disease-specific survival rates for it are more than 95%; however, it is known to poor prognosis in the advanced stage involving extrathyroid invasion and transforming to a higher grade. The current optimal guidelines for treatment and follow-up are not conclusive.

In conclusion, the extranodal marginal zone lymphoma of MALT of the thyroid gland is rare and not fully understood. In this case, despite short-term follow-up of 4 years, serial sonographic features may be meaning in the understanding of an indolent course and the knowledge of serial changes of an extranodal marginal zone lymphoma of MALT of the thyroid. Because even the clinical prognosis is worse in the advanced stage of it, patients with a focal thyroid nodule that characterized the linear echogenic strands and segmental pattern in the background of Hashimoto's thyroiditis on ultrasonography should undergo further evaluation and careful surveillance for the possibility of an extranodal marginal zone lymphoma of MALT.
